# Diagnosis of Idiopathic Pulmonary Fibrosis in High-Resolution Computed Tomography Scans Using a Combination of Handcrafted Radiomics and Deep Learning

**DOI:** 10.3389/fmed.2022.915243

**Published:** 2022-06-23

**Authors:** Turkey Refaee, Zohaib Salahuddin, Anne-Noelle Frix, Chenggong Yan, Guangyao Wu, Henry C. Woodruff, Hester Gietema, Paul Meunier, Renaud Louis, Julien Guiot, Philippe Lambin

**Affiliations:** ^1^The D-Lab, Department of Precision Medicine, GROW-School for Oncology and Reproduction, Maastricht University, Maastricht, Netherlands; ^2^Department of Diagnostic Radiology, Faculty of Applied Medical Sciences, Jazan University, Jazan, Saudi Arabia; ^3^Department of Respiratory Medicine, University Hospital of Liège, Liège, Belgium; ^4^Department of Medical Imaging Center, Nanfang Hospital, Southern Medical University, Guangzhou, China; ^5^Department of Radiology, Union Hospital, Tongji Medical College, Huazhong University of Science and Technology, Wuhan, China; ^6^Department of Radiology and Nuclear Medicine, GROW-School for Oncology, Maastricht University Medical Center, Maastricht, Netherlands; ^7^Department of Radiology, University Hospital of Liège, Liège, Belgium

**Keywords:** artificial intelligence (AI), radiomics, computed tomography, interpretability, idiopathic pulmonary fibrosis, interstitial lung disease

## Abstract

**Purpose:**

To develop handcrafted radiomics (HCR) and deep learning (DL) based automated diagnostic tools that can differentiate between idiopathic pulmonary fibrosis (IPF) and non-IPF interstitial lung diseases (ILDs) in patients using high-resolution computed tomography (HRCT) scans.

**Material and Methods:**

In this retrospective study, 474 HRCT scans were included (mean age, 64.10 years ± 9.57 [SD]). Five-fold cross-validation was performed on 365 HRCT scans. Furthermore, an external dataset comprising 109 patients was used as a test set. An HCR model, a DL model, and an ensemble of HCR and DL model were developed. A virtual *in-silico* trial was conducted with two radiologists and one pulmonologist on the same external test set for performance comparison. The performance was compared using DeLong method and McNemar test. Shapley Additive exPlanations (SHAP) plots and Grad-CAM heatmaps were used for the *post-hoc* interpretability of HCR and DL models, respectively.

**Results:**

In five-fold cross-validation, the HCR model, DL model, and the ensemble of HCR and DL models achieved accuracies of 76.2 ± 6.8, 77.9 ± 4.6, and 85.2 ± 2.7%, respectively. For the diagnosis of IPF and non-IPF ILDs on the external test set, the HCR, DL, and the ensemble of HCR and DL models achieved accuracies of 76.1, 77.9, and 85.3%, respectively. The ensemble model outperformed the diagnostic performance of clinicians who achieved a mean accuracy of 66.3 ± 6.7% (*p* < 0.05) during the *in-silico* trial. The area under the receiver operating characteristic curve (AUC) for the ensemble model on the test set was 0.917 which was significantly higher than the HCR model (0.817, *p* = 0.02) and the DL model (0.823, *p* = 0.005). The agreement between HCR and DL models was 61.4%, and the accuracy and specificity for the predictions when both the models agree were 93 and 97%, respectively. SHAP analysis showed the texture features as the most important features for IPF diagnosis and Grad-CAM showed that the model focused on the clinically relevant part of the image.

**Conclusion:**

Deep learning and HCR models can complement each other and serve as useful clinical aids for the diagnosis of IPF and non-IPF ILDs.

## Introduction

Interstitial lung disorders (ILDs) are a diverse group of ailments with an estimated 200 distinct entities and are linked with high morbidity and death (1). Many different parenchymal lung disorders have similar clinical signs and patterns of lung injury. Several disorders, including idiopathic pulmonary fibrosis (IPF), have unknown etiology and are labeled idiopathic or cryptogenic, while the rest are linked to other diseases, particularly connective tissue diseases, or to environmental exposures (2–6). One of the most common types of ILDs is IPF, a progressive illness marked by decreased lung function (7). IPF has an estimated incidence rate between 2.8 and 18 cases per 100,000 per year in Europe and North America (8). The median survival rate of patients with IPF is between 2 and 4 years from diagnosis (9). A prompt diagnosis and management are crucial for slowing down the progression of these lung disorders.

Medical imaging is becoming increasingly crucial for disease diagnosis, prognosis, and treatment planning in precision medicine (10). Computed tomography (CT) provides visual data that may be used to enhance decision-making (4, 11). However, qualitative CT evaluation remains challenging and frequently varies amongst experts (12). The diagnosis of idiopathic pulmonary fibrosis using high-resolution computed tomography (HRCT) is a difficult task and high inter-observer variability is associated with it even with experienced radiologists (13). Consequently, there is a need for an automated clinical tool that can aid clinicians for accurate and timely diagnosis.

Artificial intelligence is becoming increasingly popular due to the increasing amount of imaging data and available computational resources (14). The use of quantitative imaging techniques in medical imaging has grown at an exponential rate (15). Handcrafted radiomics (HCR) is a quantitative approach that measures and extracts high-dimensional imaging characteristics to aid clinical decision-making (15, 16). Deep learning (DL) methods learn different features and representations from the image data without the need for explicit feature engineering (17). Convolutional neural networks (CNNs) have shown remarkable results on numerous diagnostic tasks using medical image data including the diagnosis of fibrotic lung disease (18).

Despite promising results demonstrated by HCR and DL models for various medical imaging tasks, the clinical utility of such models is limited due to their lack of interpretability (19). Shapley Additive exPlanations (SHAP) (20) and Gradient-weighted class activation maps (Grad-CAM) (21) are *post-hoc* interpretability methods that are useful for understanding the decision-making process of HCR and DL models, respectively.

In this paper, we propose a machine learning-based HCR pipeline and a DL pipeline for the automated diagnosis of IPF and non-IPF ILDs patients. We also perform an in-silico trial with experienced radiologists to compare the performance of HCR and DL on a test dataset. Furthermore, we use *post-hoc* interpretability methods to aid the incorporation of these automated diagnostic tools in the clinical workflow.

## Materials and Methods

### Patients

**FIGURE 1:**
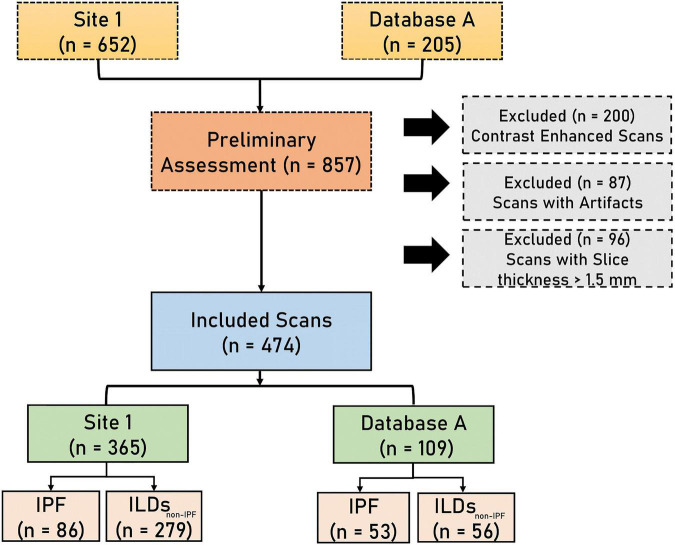
The flowchart diagram shows the patient selection process. IPF, idiopathic pulmonary fibrosis, ILDs_*non–IPF*_, non-IPF interstitial lung diseases.

A total of 652 HRCT scans were obtained from Site 1 (University Liege hospital) and 205 HRCT scans were obtained from database A [The Lung tissue research consortium database (LTCR)]. The inclusion criteria were: the availability of non-contrast enhanced HRCT and the availability of HRCT with slices thickness of less than 1.5 mm. The exclusion criteria were: the use of contrast enhancement, images containing metal or motion artifacts, and images reconstructed with a slice thickness larger than 1.5 mm. All diagnoses were confirmed by the Multidisciplinary discussion (MDD) that included a histopathologist, pulmonologist, thoracic radiologist, and rheumatologist. Lung biopsy is only required in case of ILD inconsistent with IPF. Figure 1 shows the patient selection process. Demographic data, clinical data, and measurements of pulmonary function tests (PFT) were acquired for each patient. Demographic and clinical data include age, gender, body mass index (BMI), forced expiratory volume in 1 s (FEV1), forced vital capacity (FVC), and diffusion capacity of the lungs for carbon monoxide (DLCO).

### Imaging Acquisition and Segmentation

The HRCT scans at site 1 were acquired at the same hospital using two different vendors (Siemens and GE). The scans acquired from database A were acquired using four different CT vendors (Siemens, GE, Philips, and Toshiba). The slice thickness of the scans varied between 0.5 and 1.5 mm. A further detailed description of the CT acquisition parameters can be found in Supplementary Table 1. Whole lung segmentation was performed using an automated workflow created in MIM software (MIM Software Inc., Cleveland, OH, United States).

### Data Split

Five-fold cross-validation was performed on data from Site 1 consisting of 365 HRCT scans containing 279 non-IPF ILDs, and 86 IPF patients. External data from database A, comprising 53 IPF patients and 56 non-IPF ILDs patients was used to benchmark the performance of the proposed AI tools along with the *in-silico* trial.

### Handcrafted Radiomics

#### Handcrafted Radiomics Feature Extraction

**FIGURE 2:**
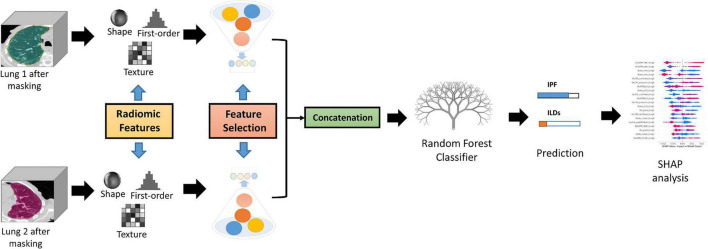
Radiomics Pipeline for Lung disease classification from CT images. The same 12 radiomics features from both lungs after feature selection are concatenated and fed to the Random Forest classifier. *Post-hoc* SHAP analysis is performed for interpretability.

To minimize the effect of the variations in image voxel size, all CT images were resampled to a 1 mm × 1 mm × 1 mm. Radiomics features were extracted from the HRCT images using the RadiomiX Discovery Toolbox^1^ which calculates handcrafted radiomics (HCR) features compliant with the Imaging Biomarkers Standardization Initiative (IBSI) (22). Voxel intensities were aggregated into 25 bins of Hounsfield Units to reduce noise and inter-scanner variability. The extracted features describe fractal dimension, intensity histogram, first-order statistics, texture, and shape. A workflow for handcrafted radiomics from segmentation to data analysis is illustrated in Figure 2.

#### Features Selection and Modeling

Features with near-zero variance (i.e., features that have the same value in ≥95% of the data points) were excluded. Then, a correlation matrix was created between all HCR features and populated using Spearman’s correlation coefficient (r). Feature pairs with |r| ≥ 0.90 were considered to be highly correlated, and the feature with the highest average correlation with all other features was removed. Furthermore, a Recursive feature elimination (RFE) using a random forest classifier was performed on the subset of features that were selected after applying Spearman’s correlation coefficient. RFE was applied with cross-validation in order to determine the accuracy of the classification and the top 12 features with the highest accuracy were selected for the final model. The same 12 features were extracted for each lung and concatenated to give a final feature vector consisting of 24 HCR features. A list of the names of the features along with their abbreviations that were used in the model can be found in Supplementary Table 2. A random forest classifier was used to construct the HCR model to predict the probability of IPF in patients using HRCTs. Random forest classifier has proven to be effective for lungs CT-based radiomics problems in recent research findings (23–25). The random forest classifier was trained with class weights of 1 for non-IPF ILDs and 3 for IPF patients to compensate for the class imbalance. Five-fold cross-validation was used for hyper-parameter tuning.

#### *Post-hoc* Interpretability

SHapley Additive exPlanations (SHAP) analysis is based on co-operative game theory (20). SHAP analysis is a *post-hoc* interpretability method that quantifies the impact of each feature on the model prediction in terms of SHAP value. SHAP summary plots provide global explanations by highlighting the effect of features on the prediction in terms of SHAP value and help in recognizing the trends. These plots show whether a high or low feature value affects the model output positively or negatively. SHAP dependence plots highlight the relationship between the model output in terms of SHAP values and the corresponding feature values. These dependence plots can be useful for quantifying the trend of model output with respect to the feature values as well as understanding the interaction effects between a pair of features.

### Deep Learning

**FIGURE 3:**
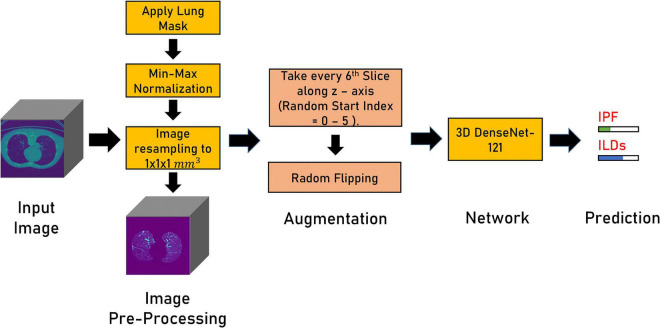
Figure shows different steps in the deep learning pipeline for the prediction of lung diseases in CT scans.

All the scans were resampled to an isotropic resolution of 1 mm × 1 mm × 1 mm. Min-max normalization was applied to the area within the lung mask. Two patches containing one lung each of size 240 ×240 ×240 voxels were extracted using the lungs masks. Both lungs were randomly flipped for augmentation and concatenated along the z-axis. The image was then downsampled by taking every sixth slice along the *z*-axis. The start index was randomly chosen in the range of 1–6. This resulted in additional augmentation and reduction of the input image size. A Densenet-121 (26, 27) classifier with 3D convolutional layers was used with weighted binary cross-entropy loss (non-IPF ILDs: 1, IPF: 3) in order to minimize the effects of data imbalance. Adam optimizer with a learning rate of 1 e^–5^ and ReduceLROnPlateau scheduler was employed. The batch size was set at 16 and the network was trained for 50 epochs. Figure 3 shows the different steps involved in training the DL model for lung disease classification in CT images.

During prediction, six input images from the test image were extracted by setting the start slice index in the range from 1 to 6 and taking every sixth consecutive slice. These six test samples are fed to the trained 3D Densenet-121 model. The final prediction is the average of the prediction of these six test samples. Heatmaps highlight the regions of the input image that the model considers important for prediction. We utilized Grad-CAM (21) heatmaps for the *post-hoc* interpretability of the Densenet-121 model.

### Ensemble Model

The ensemble methods utilize multiple machine learning methods in an effort to achieve better predictive performance as compared to the performance obtained by the constituent machine learning methods alone. We constructed an ensemble model from HCR and DL models by taking an average of the probabilities predicted by the two models.

### *In-silico* Clinical Trial

An application that allows the construction of a reference performance point by gathering medical imaging expert comments based on the visual assessment of HRCT images was created. The application allows displaying the CT images one at a time with the option of different planes (Axial, Coronal, or Sagittal), and the application also allows scrolling through the CT scan slices. The graphical user interface (GUI) of the application can be found in Supplementary Figure 1. The radiologist can select one of the two classes (IPF or ILDs other than IPF). The diagnostic performance of two radiologists (6 and 23 years of experience) and one pulmonologist (12 years of experience) was recorded for the same test dataset (*n* = 109) to perform a comparison with the machine learning-based HCR, DL, and ensemble models.

### Statistical Analysis

Statistical analysis was performed in Python (version: 3.6). Wilcoxon rank-sum test was used for the continuous variables to test the group differences and Fisher exact test for categorical variables. To assess the model’s performance, the areas under the curves (AUCs) for receiver operating characteristic (ROC) curves were compared using the DeLong test. The thresholds for each model were set at the highest Youden’s index in the training set. The performance was evaluated using accuracy, sensitivity, specificity, positive predictive value (PPV), and negative predictive value (NPV). For five-fold cross-validation, we also report the standard deviation (SD). The performance of the models on the test set was compared with the performance of clinicians using McNemar test. This study followed the Standard for Reporting Diagnostic accuracy studies (STRAD) (28) and was assessed using the Radiomics Quality Score (RQS) (29). The detailed description about RQS can be found in Supplementary Table 3.

## Results

### Patients Characteristics

A total of 474 patients, 335 of whom were diagnosed with non-IPF ILDs, and 139 with IPF, were included after the application of exclusion criteria (Figure 1). The demographic characteristics of the included patients can be found in Table 1.

**TABLE 1:** Demographic and clinical information of the study participants.

Variables	Site 1	Database A	*P*-value (*p*)
n	365	109	−⁣−
Age [mean(SD)]	64.10 (9.57)	63.61 (14.17)	0.8
Sex = M (%)	213 (87)	74 (67.9)	0.09
FEV1 [mean (SD)]	80.42 (21.47)	69.60 (20.67)	<0.001
FVC [mean(SD)]	80.52 (21.25)	67.35 (21.37)	<0.001
DLCO [mean(SD)]	51.32 (24.99)	29.84 (5.36)	<0.001
BMI [mean(SD)]	25.48 (6.45)	29.55 (5.21)	<0.001

*BMI, body mass index, FEV, forced expiratory volume, FVC, forced vital capacity, and diffusion capacity of the lungs for carbon monoxide (DLCO) are shown in the table for different patients along with their mean and standard deviation (SD).*

### Handcrafted Radiomics

**FIGURE 4:**
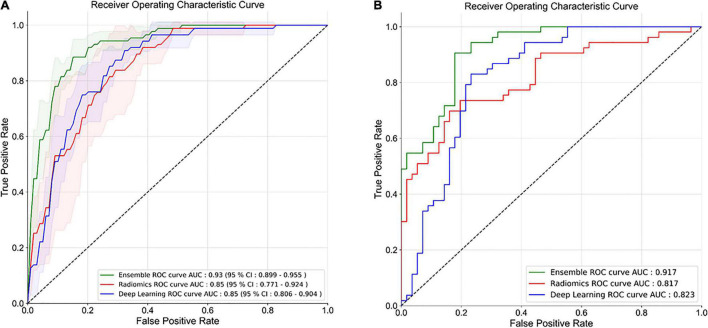
Receiver operating characteristics (ROC) curves for five-fold cross-validation **(A)** and external test dataset **(B)** for the classification of IPF and non-IPF ILDs using handcrafted radiomics (HCR), deep learning (DL), and ensemble (HCR + DL) models.

The HCR model achieved an AUC of 0.85 (95% CI: 0.771 – 0.924) in the validation set in five-fold cross-validation (Figure 4A). The threshold of 0.51 was fixed based on Youden’s index in the training set. An accuracy, sensitivity, and specificity of 0.762 ± 0.068, 0.816 ± 0.094, and 0.745 ± 0.065 were obtained in five-fold cross-validation, respectively. In the external test set, the HCR model achieved an AUC, accuracy, sensitivity, and specificity of 0.817, 0.761, 0.698, and 0.821, respectively. Tables 2, 3 show the performance metrics for the HCR model during five-fold cross-validation and external validation, respectively. Figure 4B shows the test performance for the HCR model on the external dataset. The Radiomics Quality Score (RQS) achieved for this study is 52.78% (19 of 36).

**TABLE 2:** Precision and recall metrics for five-fold cross-validation using handcrafted radiomics (HCR), deep learning (DL), and an ensemble of HCR and DL models.

Model	Accuracy	Sensitivity	Specificity	Positive predictive value (PPV)	Negative predictive value (NPV)
Handcrafted radiomics (HCR)	0.762 ± 0.068	0.816 ± 0.094	0.745 ± 0.065	0.506 ± 0.084	0.923 ± 0.040
Deep learning (DL)	0.779 ± 0.046	0.711 ± 0.10	0.800 ± 0.075	0.541 ± 0.074	0.901 ± 0.025
Ensemble (HCR + DL)	**0.852 ± 0.027**	**0.827 ± 0.005**	**0.860 ± 0.035**	**0.65 ± 0.063**	**0.94 ± 0.003**

**TABLE 3:** Comparison of diagnostic performance on the external test dataset for HCR, DL, an ensemble of HCR and DL, and *in-silico* trial with clinicians.

Model	Accuracy	Sensitivity	Specificity	Positive predictive value (PPV)	Negative predictive value (NPV)
Handcrafted radiomics (HCR)	0.761	0.698	0.821	0.787	0.741
Deep learning (DL)	0.779	0.792	0.768	0.763	0.796
Ensemble (HCR + DL)	**0.853**	**0.886**	**0.821**	**0.825**	**0.885**
*In-silico* trial with clinicians	0.66 ± 0.067	0.572 ± 0.186	0.750 ± 0.0525	0.680 ± 0.042	0.669 ± 0.100

**FIGURE 5:**
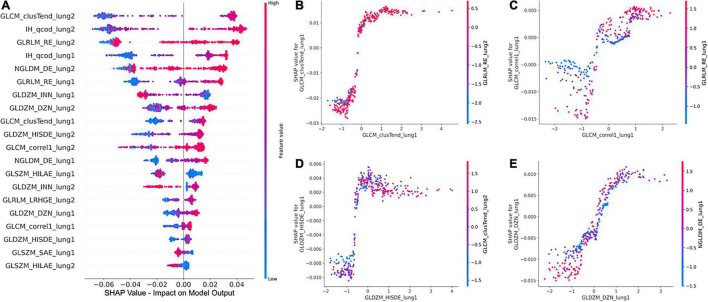
Global SHAP summary plots **(A)** demonstrate the impact of the top 20 features on the model output in terms of SHAP values and the corresponding feature values. SHAP dependence plots **(B–D)**, and **(E)** show the effect of a particular feature value on the SHAP value and its interaction with another feature.

The global SHAP summary plots in Figure 5A demonstrate that the same features extracted from each lung separately affect the model’s prediction for IPF diagnosis in a similar way. A high feature value with a positive SHAP value forces the model’s probability to be higher. The IH_qcod feature values extracted from lung1 and lung2 demonstrate a similar trend that a high feature value results in a positive SHAP value. However, there are some outliers in the trend that can seen be in features such as GLCM_correl1_lung and GLDZM_INN_lung. Similarly, the GLDZM_INN feature values extracted from lung1 and lung2 show a negative trend that a high feature value results in a negative SHAP value. Figures 5B–E show the dependence plots of GLCM_clusTend, GLCM_correl1, GLDZM_HISDE, and GLDZM_DZN features, respectively. In Figure 5C, when the feature value of GLDZM_HISDE is low, high feature values of GLCM_clusTend result in a lower SHAP value. A similar effect can be seen in Figure 5D between features GLDZM_DZN and NGLDM_DE.

### Deep Learning

The DL model achieved an AUC of 0.85 (95% CI: 0.806 – 0.904) in the validation set in five-fold cross-validation (Figure 4A). The threshold of 0.45 was fixed based on Youden’s index in the training set. An accuracy, sensitivity, and specificity of 0.779 ± 0.046, 0.711 ± 0.10, and 0.800 ± 0.075 was achieved during five-fold cross-validation, respectively. In the external test set, the DL model achieved an AUC, accuracy, sensitivity, and specificity of 0.823, 0.853, 0.886, and 0.821, respectively. Tables 2, 3 show the performance metrics for the HCR model during five-fold cross-validation and external validation, respectively. Figure 4B shows the test performance for the DL model on the external dataset.

**FIGURE 6:**
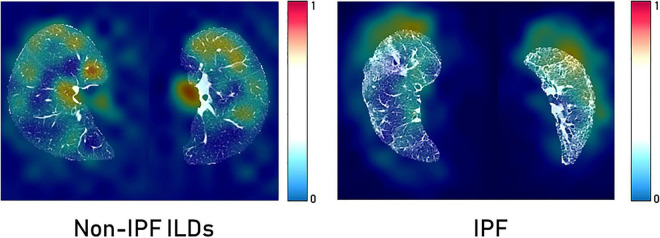
GradCAM heatmaps for *post-hoc* interpretability of IPF and non-IPF ILDs HRCT scans to understand the predictions made by the Densenet-121.

Figure 6 shows Grad-CAM overlayed on CT image slices obtained from HRCT scans from IPF and non-IPF ILDs patients. The overlayed heatmap shows the regions of the input image that the model considers important for prediction. The Grad-CAM focuses on the tissue pattern in the patient with IPF. However, no information is provided on how these areas contribute to the final model prediction.

### Ensemble

The ensemble model achieved an AUC of 0.93 (95% CI: 0.899 – 0.955) in the validation set during five-fold cross-validation (Figure 4A). The threshold of 0.49 was fixed based on Youden’s index in the training set. An accuracy, sensitivity, and specificity of 0.852 ± 0.027, 0.827 ± 0.005, and 0.860 ± 0.035 was obtained during five-fold cross-validation, respectively. In the external test set, the DL model achieved an AUC, accuracy, sensitivity, and specificity of 0.917, 0.853, 0.886, and 0.821, respectively. Tables 2, 3 show the performance metrics for the HCR model during five-fold cross-validation and external validation, respectively. The agreement between the predictions of HCR and DL models is 61.4%. The accuracy and specificity for the predictions when both the models agree were 93 and 97%, respectively. There was a statistically significant difference between the ROC curves for the ensemble model and HCR model (*p* = 0.02), and the ensemble model and the DL model (*p* = 0.005).

### *In-silico* Clinical Trials

Two radiologists and one pulmonologist achieved accuracies of 58.7, 65.1, and 75.2% with a mean of 66.3 ± 6.7% for the diagnosis of IPF and non-IPF ILDs on the external test dataset. There was a statistically significant difference between performance of the ensemble model, and that of radiologists and pulmonologists (*P* < 0.05).

## Discussion

In this study, we investigated the potential of HCR and DL to differentiate between different lung disorders i.e., IPF and non-IPF ILDs patients on HRCT scans. We also used *post-hoc* interpretability methods to explain the predictions of HCR and DL models. Moreover, we compare the performance of the proposed models to the diagnostic performance of radiologists using an *in-silico* trial on an external test set. Our results show that HCR and DL have a great potential to be used as an aid for clinical decision-making, which could minimize the time needed by radiologists, and increase diagnostic accuracy. The superior performance of an ensemble of DL and HCR models also demonstrates that these approaches can complement each other for lung disease diagnosis.

HCR and DL models achieved an accuracy of 76.2 ± 6.8% and 77.9 ± 4.6% during five-fold cross-validation, respectively. In the external test set, HCR and DL models demonstrated a similar accuracy of 76.1 and 77.9%, respectively. There was no statistically significant difference between the ROC curves for HCR and DL models. The ensemble of HCR and DL models demonstrated the best accuracy of 85.2 ± 2.7% and 85.3% for five-fold cross-validation and external test set, respectively. There was a statistically significant difference between the ROC curves for the ensemble model and HCR model (*p* = 0.023), and the ensemble model and DL model (*p* = 0.005). The HCR and DL models show an agreement of 61.4% for the predictions on the external test set. A sensitivity and specificity of 93 and 97% were obtained when both the models agreed on the prediction. Hence, HCR and DL models add complementary value to each other resulting in a boost in performance.

We compared the performance of the developed models against the performance of the radiologists using a virtual clinical trial setting. The performance of HCR (76.1%), DL (77.9%), and ensemble (85.3 %) models were better than the performance of two radiologists and one pulmonologist (66.7%) in discriminating IPF from non-IPF ILDs on the external test set. There was a statistically significant difference (*p* < 0.05) between the predictions of the ensemble model, and the two radiologists and one pulmonologist. There was a significant difference (*p* < 0.001) in the BMI, FEV, FVC, and DLCO values between site 1 and database A. The models demonstrated similar performance on the external database A despite the variability, showing that the trained models are robust and generalize well.

The clinical translation of HCR and DL is limited due to the “black-box” nature of the underlying complex classifiers. It is difficult for clinicians to understand the underlying mechanisms that govern the decision-making process of these complex classifiers. SHAP *post-hoc* explanations discover the patterns of the complex classifiers and increase transparency. SHAP global summary plots showed that Gray-level Co-occurrence Matrix Cluster Tendency and Intensity Histogram quartile coefficient of dispersion are the most important features for IPF diagnosis. These plots also showed that the same features extracted from different lungs demonstrate a similar trend in SHAP impact value. SHAP dependence plots demonstrated the effect of a single feature value and the interaction between a pair of features on the model output. Grad-CAM heatmaps highlight the area that the DL model considers important for the final prediction. These heatmaps can reinforce the trust in the model predictions if the model is focusing on the area relevant to the clinical task. However, Grad-CAM heatmaps do not offer any explanation of how the highlighted area contributes to the final prediction. Although DL demonstrates good performance, it is more opaque in nature due to its complexity that might hinder its clinical adoption.

Some studies previously investigated the potential of HCR and DL algorithms to classify lung disorders. Walsh el al. (18) employed a DL algorithm on a dataset of 1157 HRCT images for the diagnosis of fibrotic lung disease. The algorithm performance was compared to that of 91 radiologists and revealed an accuracy of 73.3%, compared to the radiologist’s median accuracy of 70.7%. When compared to Walsh et al. (18), our study demonstrated greater accuracy using HCR (76.1%), DL (77.9%), and an ensemble of HCR and DL (85.3%). Christe et al. (30) conducted another study in which they employed a computer-aided diagnostic (CAD) system (INTACT system) to diagnose IPF cases based on HRCT images and compared the performance of the CAD system to the performance of radiologists. Their findings showed that the two radiologists and the CAD system obtained an accuracy of 60, 54, and 56%, respectively. Mean RQS score of 20.4, 26.1, and 27.4% were obtained after recent analyses of papers reporting radiomics studies (31–33). This shows that RQS is a stringent and demanding criterion (34–36) that aims to encourage the best scientific practice. An RQS of 52.78% shows that this study tries to adhere to the best scientific practices and reporting guidelines.

This study has some limitations. The datasets utilized for this study contain HRCT scans acquired with different CT acquisition and reconstruction settings that can influence HCR feature values (37). Hence, phantom studies to evaluate the reproducibility of the HCR features or harmonization investigations need to be carried out to make a more robust HCR pipeline (38). Grad-CAMs only highlight the region of the input image that the model considers important for the decision-making process. There is a need to utilize interpretability methods that give an insight into how the relevant region contributes to the decision-making process (19). The high performance of an ensemble of HCR and DL model shows that these two approaches add complementary values. It may be useful to employ an interpretability method such as concept attribution that will investigate the HCR features that the DL model considers important for classification (39). A prospective virtual *in-silico* trial in a real-world environment where the predictions of DL/HCR model and *post-hoc* interpretability plots are made available to the doctors during diagnosis should be carried out to confirm the clinical utility of the proposed methods. The quality of lung segmentation can affect the performance of the models. Therefore, it is important to ensure the quality of the automatic segmentation in the presence of variability such as noise and artifacts.

At the moment, there is little research on the diagnosis of ILDs using HCR and DL. The reported results are encouraging and highlight the significant potential of HCR and DL methods for the diagnosis of IPF. In the future, HCR and DL approaches may be expanded to include treatment decisions. More studies should be conducted to explore the development of IPF at baseline and follow-up, as well as to assess the efficacy of anti-fibrotic treatment.

## Conclusion

In this study, we developed handcrafted radiomics and deep learning models for the classification of IPF and non-IPF ILDs using HRCTs. In addition, we compared the performance of both models to radiologists on an external test dataset. HCR, DL, and ensemble models demonstrated better accuracy than radiologists in a virtual *in-silico* clinical trial setting. An ensemble of HCR and DL models demonstrated the best performance highlighting the complementary value of the two quantitative approaches for lung disease diagnosis. SHAP and GRAD-CAM *post-hoc* interpretability methods are useful for explaining the predictions made by radiomics and DL models, respectively. These automated diagnostic tools can serve as a useful clinical aid for diagnosing different lung diseases.

## Data Availability Statement

The datasets presented in this article are not readily available because the data from University Liege Hospital is privately owned while data from the Lung Tissue Research Consortium database (LTCR) is publically available. Requests to access the datasets should be directed to JG, j.guiot@chuliege.be; The Lung tissue research consortium database (LTCR): https://ltrcpublic.com.

## Ethics Statement

The studies involving human participants were reviewed and approved by the Local Ethics Committee of Hospitalo Facultaire Universitaire de Liège (CHU Hospital of Liège, Belgian number: B707201422832; ref: 2022/20). The ethics committee waived the requirement of written informed consent for participation.

## Author Contributions

TR and ZS: conceptualization, methodology, formal analysis, data curation, writing – original draft, and project administration. A-NF: conceptualization. CY and GW: data curation. HW: writing – review and editing. HG, PM, and RL: resources. JG and PL: conceptualization, methodology, writing – review and editing, project administration, funding, and supervision. PL: guarantor for the manuscript. All authors contributed to the article and approved the submitted version.

## Conflict of Interest

PL reports, as non-practicing MD, in the last 3 years, within and outside the submitted work, grants/sponsored research agreements from Radiomics SA, Convert Pharmaceuticals and LivingMed Biotech. PL received a presenter fee (in cash or in kind) and/or reimbursement of travel costs/consultancy fee (in cash or in kind) from Radiomics SA, BHV, Varian, Elekta, ptTheragnostic/DNAmito, BMS, and Convert pharmaceuticals. PL has minority shares in the companies Radiomics SA, Convert pharmaceuticals, Comunicare, and LivingMed Biotech, and he is co-inventor of two issued patents with royalties on radiomics (PCT/NL2014/050248 and PCT/NL2014/050728), licensed to Radiomics SA; one issued patent on mtDNA (PCT/EP2014/059089), licensed to ptTheragnostic/DNAmito; one non-issued patent on LSRT (PCT/P126537PC00), licensed to Varian; three non-patented inventions (softwares) licensed to ptTheragnostic/DNAmito, Radiomics SA and Health Innovation Ventures and two non-issued, non-licensed patents on Deep Learning-Radiomics (N2024482, N2024889). PL confirms that none of the above entities or funding sources were involved in the preparation of this manuscript. JG reports personal fees for advisory board, work and lectures from Boehringer Ingelheim, Janssens, SMB, GSK, Roche and Chiesi, non-financial support for meeting attendance from Chiesi, Roche, Boehringer Ingelheim and Janssens. He is in the permanent SAB of Radiomics (Oncoradiomics SA) for the SALMON trial without any specific consultancy fee for this work. He is co-inventor of one issued patent on radiomics licensed to Radiomics (Oncoradiomics SA). He confirms that none of the above entities or funding was involved in the preparation of this work. HW has minority shares in the company Radiomics SA. The remaining authors declare that the research was conducted in the absence of any commercial or financial relationships that could be construed as a potential conflict of interest.

## Publisher’s Note

All claims expressed in this article are solely those of the authors and do not necessarily represent those of their affiliated organizations, or those of the publisher, the editors and the reviewers. Any product that may be evaluated in this article, or claim that may be made by its manufacturer, is not guaranteed or endorsed by the publisher.

## Footnotes

1
https://www.radiomics.bio
