# Supplementary material for: Diagnosis of Idiopathic Pulmonary Fibrosis in High-Resolution Computed Tomography Scans Using a Combination of Handcrafted Radiomics and Deep Learning
Source: Front Med (Lausanne). 2022 Jun 23;9:915243. doi: 10.3389/fmed.2022.915243 (PMC9259876; doi:10.3389/fmed.2022.915243)
Supplement: Supplementary file 1 [file Data_Sheet_1.docx]

Supplementary Material

**Table E1:** Overview of acquisition parameters of all CT images included in the study

| **Parameters** | **Center 1** | **Database A** |
| --- | --- | --- |
| **Manufacturer** | Siemens, GE | Siemens, GE, Toshiba, Philips |
| **Tube voltage** | 80-140 | 120-140 |
| **Tube current** | Automatic | Automatic |
| **Pixel spacing** | 0.58 - 0.97 mm^2^ | 0.52-0.93 mm^2^ |
| **Reconstruction kernel** | B20f, B20s, B30f, B30s, B31f, B50f, B50s, B60s, B70f, Bone  , Br36f, Lung, STANDARD, Bf37f, Br32f, Br34f, Br36f, Br58f  , Br59f, FC02, FC51 | B46f, B60f, B50f, B41f, B31f, B60s  , B70s, B40f, FC52, C, Lung, Bone, STANDARD |
| **Slice Thickness** | 0.62 mm, 1 mm, 1.25 mm, 1.5 mm, 0.5 mm | 0.62 mm, 0.75 mm, 1 mm, 1.25 mm, 1.5 mm, 2.5 mm, 3 mm, 5 mm |
| **Matrix** | 512x512 | 512x512 |

**Table E2**: A list of features names used in the model.

| **Feature Abbreviation** | **Feature name** |
| --- | --- |
| **GLCM_clusTend** | Gray-level Co-occurrence Matrix_Cluster Tendency |
| **GLCM_correl1** | Gray-level Co-occurrence Matrix_Correlation |
| **GLDZM_DZN** | Gray-Level Distance_Zone Matrix_Distance_Zone Non-uniformity |
| **GLDZM_HISDE** | Gray-Level Distance_Zone Matrix_High Intensity Small Distance Emphasis |
| **GLDZM_INN** | Gray-Level Distance_Zone Matrix_Intensity Non-uniformity Normalized |
| **GLRLM_LRHGE** | Gray-Level Run-Length Matrix_Long Run High Gray-level Emphasis |
| **GLRLM_RE** | Gray-Level Run-Length Matrix_Run Entropy |
| **GLSZM_HILAE** | Gray-Level Size-Zone Matirx_High Intensity Large Area Emphasis |
| **GLSZM_SAE** | Gray-Level Size-Zone Matirx_Small Area Emphasis |
| **IH_qcod** | Intensity Histogram quartile coefficient of dispersion |
| **NGLDM_DE** | Neighboring Gray-Level Dependence Matirix_Dependence Entropy |
| **Stats_min** | First-order statistics_minimum |

**Figure E1:** The Graphical User Interface of the application used for conducting the *in-silico* trial.


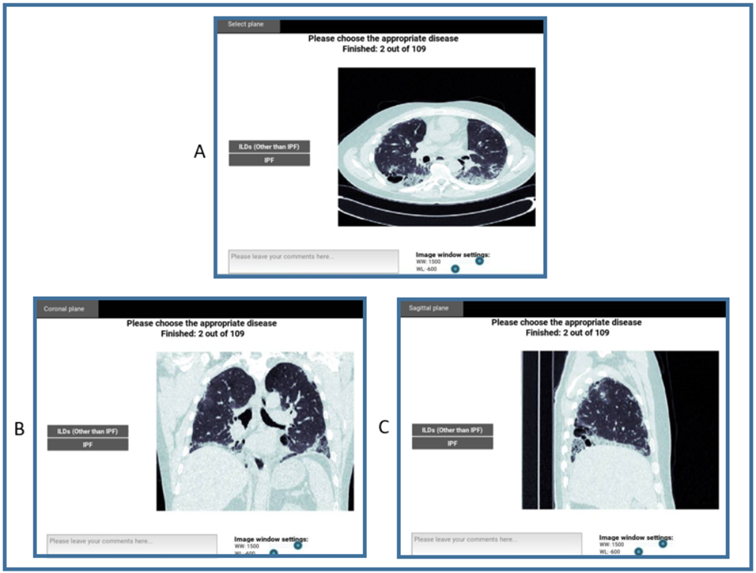


**Table E3**: Radiomics Quality Score Calculation.

| Counts | Criteria | Points | Score |
| --- | --- | --- | --- |
| 1 | Image protocol quality - well-documented image protocols (for  example, contrast, slice thickness, energy, etc.) and/or usage of public  image protocols allow reproducibility/replicability | + 1 (if protocols are well-documented) + 1 (if public protocol is used) | 1 |
|  |  |  |  |
| 2 | Multiple segmentations - possible actions are: segmentation by  different physicians/algorithms/software, perturbing segmentations  by (random) noise, segmentation at different breathing cycles. Analyse  feature robustness to segmentation variabilities | 1 | 1 |
|  |  |  |  |
| 3 | Phantom study on all scanners - detect inter-scanner differences  and vendor-dependent features. Analyse feature robustness to these  sources of variability | 1 | 0 |
|  |  |  |  |
| 4 | Imaging at multiple time points - collect images of individuals at  additional time points. Analyse feature robustness to temporal  variabilities (for example, organ movement, organ expansion/  shrinkage) | 1 | 0 |
|  |  |  |  |
| 5 | Feature reduction or adjustment for multiple testing - decreases the  risk of overfitting. Overfitting is inevitable if the number of features  exceeds the number of samples. Consider feature robustness when  selecting features | - 3 (if neither measure is implemented) + 3 (if either measure is  implemented) | 3 |
|  |  |  |  |
| 6 | Multivariable analysis with non radiomics features (for example, EGFR  mutation) - is expected to provide a more holistic model. Permits  correlating/inferencing between radiomics and non radiomics features | 1 | 0 |
|  |  |  |  |
| 7 | Detect and discuss biological correlates - demonstration of phenotypic  differences (possibly associated with underlying gene–protein  expression patterns) deepens understanding of radiomics and biology | 1 | 0 |
|  |  |  |  |
| 8 | Cut-off analyses - determine risk groups by either the median, a  previously published cut-off or report a continuous risk variable.  Reduces the risk of reporting overly optimistic results | 1 | 0 |
|  |  |  |  |
| 9 | Discrimination statistics - report discrimination statistics (for example,  C‑statistic, ROC curve, AUC) and their statistical significance  (for example, p‑values, confidence intervals). One can also apply  resampling method (for example, bootstrapping, cross-validation) | + 1 (if a discrimination statistic and its statistical significance are  reported) + 1 (if a resampling method technique is also applied) | 1 |
|  |  |  |  |
| 10 | Calibration statistics - report calibration statistics (for example,  Calibration-in‑the-large/slope, calibration plots) and their statistical  significance (for example, P‑values, confidence intervals). One  can also apply resampling method (for example, bootstrapping,  cross-validation) | + 1 (if a calibration statistic and its statistical significance are  reported) + 1 (if a resampling method technique is also applied) | 1 |
|  |  |  |  |
| 11 | Prospective study registered in a trial database - provides the highest  level of evidence supporting the clinical validity and usefulness of the  radiomics biomarker | + 7 (for prospective validation of a radiomics signature in an  appropriate trial) | 7 |
|  |  |  |  |
| 12 | Validation - the validation is performed without retraining and without  adaptation of the cut-off value, provides crucial information with  regard to credible clinical performance | - 5 (if validation is missing) + 2 (if validation is based on a dataset  from the same institute) + 3 (if validation is based on a dataset from  another institute) + 4 (if validation is based on two datasets from two  distinct institutes) + 4 (if the study validates a previously published  signature) + 5 (if validation is based on three or more datasets from  distinct institutes)  *Datasets should be of comparable size and should have at least  10 events per model feature | 3 |
|  |  |  |  |
| 13 | Comparison to ‘gold standard’ - assess the extent to which the model  agrees with/is superior to the current ‘gold standard’ method (for  example, TNM-staging for survival prediction). This comparison shows  the added value of radiomics | 2 | 2 |
|  |  |  |  |
| 14 | Potential clinical utility - report on the current and potential application  of the model in a clinical setting (for example, decision curve analysis). | 2 | 0 |
|  |  |  |  |
| 15 | Cost-effectiveness analysis - report on the cost-effectiveness of the  clinical application (for example, QALYs generated) | 1 | 0 |
|  |  |  |  |
| 16 | Open science and data - make code and data publicly available. Open  science facilitates knowledge transfer and reproducibility of the study | + 1 (if scans are open source) + 1 (if region of interest  segmentations are open source) + 1 (if code is open source)  + 1 (if radiomics features are calculated on a set of representative  ROIs and the calculated features and representative ROIs are | 0 |
| RQS Sum = 19 out of 36 | | | |
| RQS = 52.78 % | | | |
